# Synthesis and Docking Studies of 2,4,6-Trihydroxy-3-Geranylacetophenone Analogs as Potential Lipoxygenase Inhibitor

**DOI:** 10.3390/molecules190811645

**Published:** 2014-08-05

**Authors:** Chean Hui Ng, Kamal Rullah, Mohd Fadhlizil Fasihi Mohd Aluwi, Faridah Abas, Kok Wai Lam, Intan Safinar Ismail, Radhakrishnan Narayanaswamy, Fadzureena Jamaludin, Khozirah Shaari

**Affiliations:** 1 Laboratory of Natural Products, Institute of Bioscience, Universiti Putra Malaysia (UPM), Selangor Darul Ehsan, 43400 UPM Serdang, Malaysia; E-Mails: nch_chean@hotmail.com (C.H.N.); faridah_abas@upm.edu.my (F.A.); safinar@upm.edu.my (I.S.I.); nrkishnan@gmail.com (R.N.); 2 Faculty of Pharmacy, Universiti Kebangsaan Malaysia (UKM), Jalan Raja Muda Abdul Aziz, 50300 Kuala Lumpur, Malaysia; E-Mails: kamalrullah@yahoo.co.id (K.R.); fadhfasihi@yahoo.com (M.F.F.M.A); david_lam_98@yahoo.com (K.W.L.); 3 Department of Food Science, Faculty of Food Science and Technology, Universiti Putra Malaysia (UPM), Selangor Darul Ehsan, 43400 Serdang, Malaysia; 4 Forest Research Institute (FRIM), Selangor Darul Ehsan, 52109 Kepong, Malaysia; E-Mail: fadzureena@frim.gov.my

**Keywords:** lipoxygenase, analogs, Friedel-Craft acylation, Friedel-Craft alkylation, molecular docking

## Abstract

The natural product molecule 2,4,6-trihydroxy-3-geranyl-acetophenone (tHGA) isolated from the medicinal plant *Melicope ptelefolia* was shown to exhibit potent lipoxygenase (LOX) inhibitory activity. It is known that LOX plays an important role in inflammatory response as it catalyzes the oxidation of unsaturated fatty acids, such as linoleic acid to form hydroperoxides. The search for selective LOX inhibitors may provide new therapeutic approach for inflammatory diseases. Herein, we report the synthesis of tHGA analogs using simple Friedel-Craft acylation and alkylation reactions with the aim of obtaining a better insight into the structure-activity relationships of the compounds. All the synthesized analogs showed potent soybean 15-LOX inhibitory activity in a dose-dependent manner (IC_50_ = 10.31–27.61 μM) where compound **3e** was two-fold more active than tHGA. Molecular docking was then applied to reveal the important binding interactions of compound **3e** in soybean 15-LOX binding site. The findings suggest that the presence of longer acyl bearing aliphatic chain (5Cs) and aromatic groups could significantly affect the enzymatic activity.

## 1. Introduction

Lipoxygenases (LOXs) are a large monomeric protein family with non-heme, non-sulphur, and iron cofactor containing dioxygenases that catalyze the polyunsaturated fatty acid (PUFA) as a substrate to yield hydroperoxides [1]. LOXs can be found in both plants and animals, while the isoenzymes of LOX can be distinguished based on the peroxidation site of the substrate [2,3]. LOXs from plants and mammals have highest level of sequence identity in the area of catalytic domain containing non-heme iron atom [2]. The important mammalian LOXs are 5-, 8-, 12-, and 15-LOX, while the plant LOXs are 5- and 15-LOX. The common substrates of animals (arachidonic acid, 20-carbon) and plants (linoleic and α-linoleic acids, 18-carbon) differ in their chain lengths [4]. Mammalian LOXs catalyze the conversion of arachidonic acid to form hydroperoxy eicosatetraenoic acids (HPETEs) via radical mechanism [5]. LOX from plants oxygenate linoleic acid to generate 13-hydroperoxy-9(*Z*),11(*E*)-octadecadienoic acid (13-HPOD) [6].

The biological properties of human LOXs had been widely studied due to their involvement in several diseases. The 5-LOX pathway is the source of potent pro-inflammatory mediators [7]. Leukotrienes (LTs) are important mediators of allergic asthma produced through 5-LOX pathway. Cysteinyl leukotriene (cysLT), the metabolite of leukotrienes (LTs) acts as a bronchoconstrictor that can trigger asthmatic attack [3,8]. Recently, 15-LOX has been said to be involved in the progression of cancer [9,10] and chronic obstructive pulmonary disease (COPD) [9]. Furthermore, the immediate products from the oxidation of arachidonic acid and linoleic acid by 15-LOX have been shown to be pro-inflammatory [11] and pro-thrombotic [12]. Due to difficulties in obtaining the human enzyme in sufficiently purified form, and the availability of soybean enzyme, many researches have employed soybean 15-LOX [13] as a biological screen in discovering new LOX inhibitors.

Our earlier studies on the anti-inflammatory properties of the medicinal plant *Melicope ptelefolia* resulted in the identification of 2,4,6-trihydroxy-3-geranylacetophenone (tHGA), a drug-like compound containing the phloroglucinol structural-core, as the bioactive principle [14,15]. Initially, this compound was found to exert a dose-dependent inhibition against soybean 15-LOX with an IC_50_ value of 20 μM. In addition, this compound was subsequently shown to exert a dose-dependent inhibition of cysteinyl leukotriene secretion from activated macrophage cells. Further exploration of both the chemistry and pharmacology of tHGA revealed that tHGA inhibited 5-lipoxygenase (5-LOX) and both cyclooxygenase (COX) isoforms, albeit with greater selectivity towards COX-2 [16,17]. When used in an acute model of murine asthma, tHGA was as effective as Zileuton, a commercial LOX inhibitor, in controlling airway hyperresponsiveness to methacholine challenge, reducing pulmonary cellular infiltration, goblet cell metaplasia, cytokine (IL-4, IL-5, IL-13) and cysteinyl leukotriene secretion and systemic IgE concentrations [16,17,18]. 

**Figure 1:**
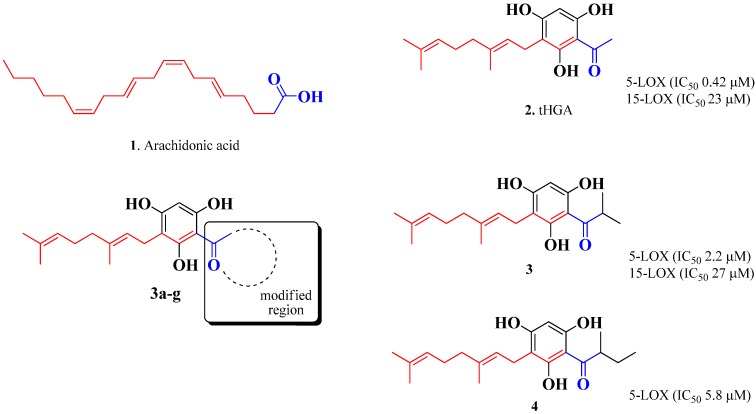
IC_50_ values of naturally active LOX inhibitors in which the structure features is similar to that of arachidonic acid.

The acylphloroglucinol group of natural products is prolific in terms of exerting many interesting biological properties [19]. As a class, these compounds are based on an aromatic ring that in many cases may exhibit keto-enol tautomerism. The phloroglucinol scaffold is substituted with simple to complex isoprene side chains and a simple acyl group. As in the case of tHGA, those carrying an acetyl group are also referred to as acetophenones. Two acylphloroglucinols namely 3-geranyl-1-(2'-methylpropanoyl)phloroglucinol (IC_50_ = 2.2 μM) and 3-geranyl-1-(2'-methylbutanoyl)phloroglucinol (IC_50_ value = 5.8 μM), which were isolated from *Hypericum empetrifolium* exhibited strong inhibitory activity against 5-LOX (Figure 1) [20].

Interestingly, we have observed that arachidonic acid and the three isolated compounds (tHGA, 3-geranyl-1-(2'-methylpropanoyl)phloroglucinol and 3-geranyl-1-(2'-methylbutanoyl)phloroglucinol), as shown in Figure 1, have similar structural features in terms of having a hydrophilic region and a hydrophobic region. As in these three isolated compounds, a combination of the phloroglucinol core structure with a hydrophilic acyl group and a hydrophobic geranyl group exhibited great pharmacological activity. The observed inhibitory action of these compounds on LOX indicated that the lipophilic geranyl group is important for the pharmacological activity. The only difference between these compounds is the nature of the acyl substituent. This motivated us to carry out the synthesis of tHGA analogs by varying the acyl substituent but preserving the geranyl group.

All the synthesized compounds were tested for their *in vitro* anti-inflammatory activities against soybean 15-LOX. Soybean 15-LOX assay can be used as a preliminary study of human 15-LOX, due to the similarities in their structure and mechanism [21]. We further carried out molecular docking studies to obtain a better insight about the structure-activity relationships (SARs) of the compounds. This study will help to identify important structural features that influence the ligand-protein interactions between the tHGA analogs and the enzyme.

## 2. Results and Discussion

### 2.1. Synthesis of tHGA Analogs

**Figure 2:**
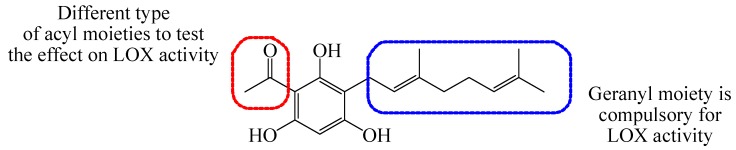
Modification on acyl moiety on the structure of tHGA.

Previously, our group has successfully synthesized tHGA and evaluated it for LOX inhibitory activity [14,15,17,18]. Our strategy was straightforward which is to modify the acyl group of tHGA structure while preserving the geranyl moiety that resembles the structure of hydrophobic tail of arachidonic acid (Figure 2).

In this study, we would like to report the synthesis of 3-geranyl-1-(2'-methylpropanoyl)phloroglucinol (**3a**), a natural compound found in *Hypericum empetrifolium*. This compound was also reported previously to be a 5-LOX inhibitor from *Hypericum empetrifolium* [20]*.* To date, there has been no report regarding its effect on soybean 15-LOX activity. Briefly, direct Friedel-Craft acylation of phloroglucinol (**1**) was carried out using isobutyryl chloride as the acylating agent in the presence of anhydrous aluminum chloride as the solid catalyst to yield compound **2a** as described by Hartl and Reininger (1977) [22]. Subsequently, the geranyl moiety was introduced via electrophilic substitution of geranyl bromide in the presence of anhydrous potassium carbonate as base in dry methanol and refluxed for eight hours to give the target compound **3a** in moderate yield (19.5%) [18].

**Scheme 1:**
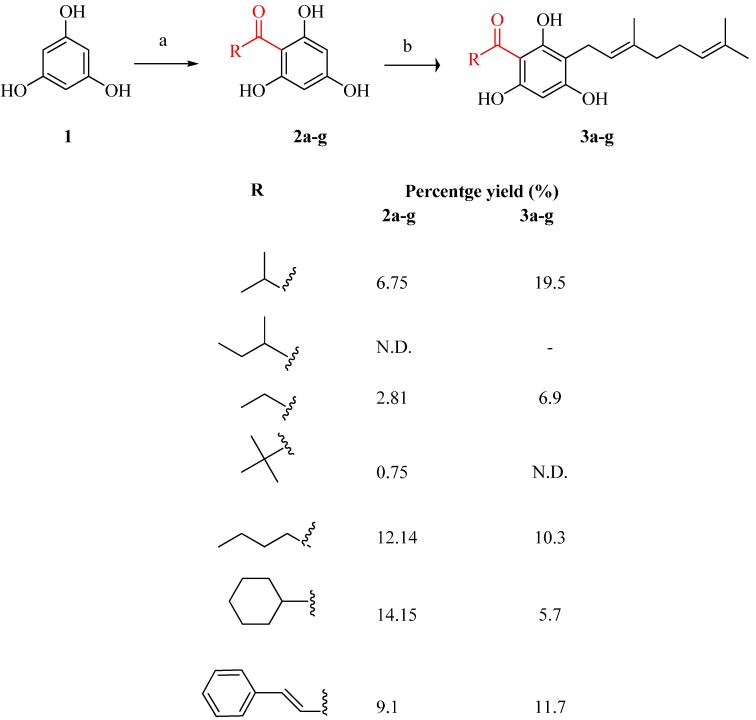
Reagents and conditions: (**a**) Acyl chloride, anhydrous aluminum chloride, hydrochloric acid, dichloromethane; (**b**) Geranyl bromide (C_10_H_17_Br), anhydrous potassium carbonate, dry methanol, reflux 8 h.

Other tHGA analogs were also prepared, according to the general reaction scheme as illustrated in Scheme 1. The percentage yield of the compounds were also reported in Scheme I. Aliphatic straight chain acyl halide (propionyl and pentanoyl chloride) and branched chain acyl halide (2-methylbutanoyl and pivaloyl chloride) were introduced to the phloroglucinol core structure using the same method with the preparation of **2a** to afford intermediates **2c**, **2e**, **2b** and **2d**. In order to expand the structure-activity relationship (SAR) scope, the cyclic ring and aromatic ring acyl substituent were also introduced to yield **2f** and **2g** to study the different effects of the acyl substituents on LOX activity. The intermediates (**2b**–**g)** were then further reacted with geranyl bromide to afford target analogs (**3b**–**g**). However, we did not manage to isolate compound **2b** and **3d** due to purification problem.

The methodology for Friedel-Craft acylation and alkylation are shown in the methodology section. The chemical structures of the synthesized compounds are shown (Figure S1 and S2, see Supplementary data). All of the synthesized compounds were characterized by using Nuclear Magnetic Resonance (NMR) Spectroscopy and Mass Spectrometry (MS) (Supplementary data Figure S3–S35).

N.D.: Not Determined, due to purification problem.

### 2.2. In Vitro Soybean 15-Lipoxygenase Inhibition Assay

The target compounds (**3a**, **3c**, **3e**–**g**), intermediates (**2a**, **2c**–**g**) and the starting material (**1**) were preliminary evaluated *in vitro* for their ability to inhibit soybean 15-LOX activity by using spectrophotometric method [17]. Bioactivity screening for soybean 15-LOX inhibition by the target compounds was carried out at 100 μg/mL concentration. Nordihydroguaiaretic acid (NDGA), a known soybean 15-LOX inhibitor was used as reference standard. The bioactivities of the compounds were also compared to tHGA as the parent compound.

The screening results (Table 1) revealed that phloroglucinol (**1**) and phloracetophenones (**2a**, **2d**–**f**) demonstrated weak inhibition of soybean 15-LOX, with values ranging between 16% and 24% at 100 μg/mL concentration. Intermediates **2c** and **2g** did not exhibit any anti-inflammatory activity against the enzyme. Meanwhile the geranylated compounds (**3a**, **3c**, **3e**–**g**) demonstrated moderate to excellent anti-inflammatory activities in a dose-dependent manner with IC_50_ ranging from 10.31 to 27.61 μM. The IC_50_ value of NDGA (reference compound), tHGA and its analogs with intermediates are shown in Table 1. It is noted that the IC_50 _ value of tHGA (parent compound) was consistent with our previous study [17]. Interestingly, the three target compounds (**3c**, **3e** and **3g**) exhibited better soybean 15-LOX inhibition with improvement in activities ranging from approximately 30%–50%.

**Table 1:** Anti-inflammatory activities of phloroglucinol (**1**), phloracetophenones (**2a**, **2c**–**g**), geranylacetophenones (**3a**, **3c**, **3e**–**g**), 2,4,6-trihydroxy-3-geranylacetophenone (tHGA) and Nordihydroguaiaretic acid (NDGA) on soybean 15-LOX enzyme.

Compound	% Inhibition (100 μg/mL)	IC_50_ Value (μM) Mean ± SEM
**1**	23.2 ± 2.2	>100
**2a**	19.4 ± 5.0	>100
**2c** *	n.a	>100
**2d**	16.9 ± 1.9	>100
**2e**	24.5 ± 3.2	>100
**2f**	17.5 ± 3.0	>100
**2g** *	n.a	>100
**3a** ^Ω^	90.3 ± 4.1	27.61 ± 3.6
**3c**	94.4 ± 3.0	12.32 ± 0.6
**3e** ^#^	90.3 ± 4.4	10.31 ± 1.5
**3f**	88.8 ± 5.9	26.31 ± 1.3
**3g**	90.8 ± 7.6	15.20 ± 1.2
tHGA **	94.3 ± 3.4	23.61 ± 1.7
NDGA ***	100.0 ± 0.0	0.10 ± 0.0

* n.a is not active; ^#^ most active compound; ** tHGA as parent compound; *** NDGA as reference compound; ^Ω^ Synthesized compound that having same structure with isolated compound from *Hypericum empetrifolium*.

**Figure 3:**
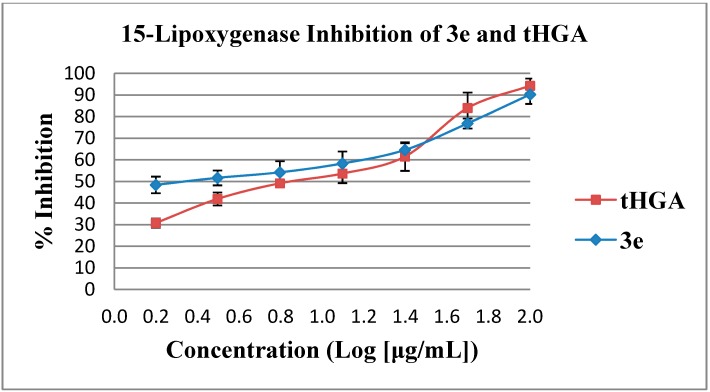
Inhibition of soybean 15-LOX by compound **3e** compared to tHGA.

The non-geranylated intermediates (**2a**, **2d**–**f**) exhibited weak inhibitory activities (>100 μM), while the preservation of the geranyl moiety as in compounds (**3a**, **3c**, **3e**–**g**) improved the enzyme inhibitory activities. In terms of SARs, compound **3a** is slightly less potent when compared to tHGA. However, the removal of a branched methyl group as in compound **3c** resulted in a two-fold increment in activity in comparison with tHGA. Elongation of the aliphatic chain length of the acyl group to 5Cs as in compound **3e** yielded the most potent inhibitor in this series (IC_50_ = 10.31 μM ± 1.5). The dose response curves of compound **3e** and tHGA are showed in Figure 3. Our present findings are in agreement with another study by Kubo and coworkers on alkyl protocatechuates [23]. The authors reported that the LOX inhibitory activity increases with increasing lipophilicity arising from longer alkyl chain lengths of their synthesized compounds. The replacement of aliphatic chain by cyclohexyl ring significantly decreased the inhibitory activity. This might be due to the cyclohexyl ring is no longer flat and the bulkier structure make the binding region for the cyclic ring become narrow and thus making the analog incapable of fitting into the active site [24]. In contrast, the replacement of cyclohexyl ring with a longer aromatic ring exhibited better inhibitory activity than tHGA since the aromatic ring is planar enough to fit into the active site. Among the five active compounds, three compounds **3c**, **3e** and **3g** exhibited higher inhibitory activities than tHGA.

### 2.3. Molecular Docking

Lipoxygenases (LOXs) are unique because of their iron cofactor which is a single ion bound by six side chains of histidine residues and the oxygen of carboxylic group of the C-terminus [25,26]. Some inhibitors have been reported to bind either directly or indirectly to the adjacent amino acid residues of Fe^3+^ cofactor [25]. Three known LOX inhibition mechanism has been reported to date including (i) redox inhibitors, which disturb the redox cycle of LOX, (ii) iron-chelating agents, and (iii) competitive inhibitors, which compete with substrate to bind to the enzyme active site [27]. In this study, first the protein structure (**1IK3**: 2.0 Å) was retrieved from Brookhaven Protein Data Bank (PDB). In order to investigate the orientation of the most active compound **3e** in the binding site of fatty acid, Discovery Studio^®^ 3.1 (Accelrys, SD, USA) was employed for the docking simulation and analysis. All the ligands except for the cofactor iron (Fe^3+^) were removed from the active pocket site of the protein crystal structure in order to study the inhibition mechanism of the active compound.

**Figure 4:**
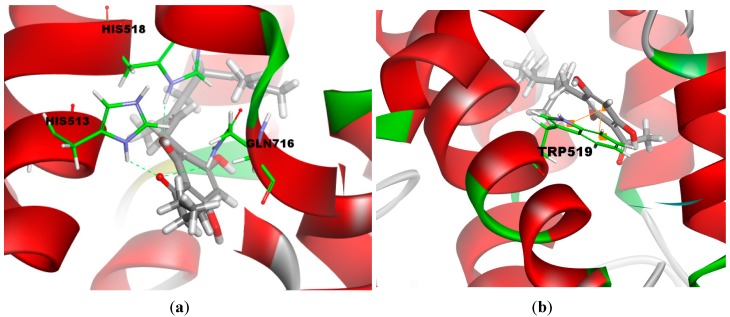
Three-dimensional (3-D) docking model of binding interaction of the compound with amino acid residues: (**a**) Compound **3e**; (**b**) tHGA; The atom colouring for the compounds is the following: carbons in grey, oxygen in red, nitrogen in blue, hydrogen in white and amino acid in green color. The orange line indicates the π-interaction, while the green line indicates the hydrogen-bonding interactions.

In terms of cDOCKERS interaction energy, compound **3e** **(**−44.51 kcal/mol) was predicted to exhibit a more favorable binding interaction energy than tHGA (−35.10 kcal/mol). According to the docking results, compound **3e** interacts with three amino acid residues (Figure 4a). The oxygen atom from the carbonyl group of acyl moiety forms hydrogen bond with His513 (C=O----H-N with distance 2.3 Å) and Gln716 (C=O----H-N with distance 2.5 Å), respectively. Furthermore, the hydroxyl group from the phloroglucinol moiety forms important hydrogen bond with His518 (H-O----H-N with distance 2.1 Å). In contrast, the aromatic ring of tHGA only involved in a weak π-π interaction with indole ring of Trp519 (aromatic ring----aromatic indole with distances 5.4 Å and 5.6 Å) (Figure 4b). This observation correlates well with the bioassay results, in which compound **3e** exhibited higher potency than tHGA as it participates in strong hydrogen bonding interactions with the aforementioned amino acid residues. As shown in Supplementary data Figure S36 , compound **3g** was predicted to bind with three amino acid residues. Apart from Asp766 (O-H----O=C with distance 2.4 Å), compound **3g** was predicted to bind similarly as compound **3e**. However, judging from docking results, compound **3g** (−35.81 kcal/mol) was found to exhibit lower cDOCKER interaction energy than **3e**(−44.51 kcal/mol). It is highly suggestive that steric effects imposed by the aromatic ring could be the major culprit behind the reduction in LOX inhibition. The bioassay results presented in the table above correlates well with our hypothesis.

**Figure 5:**
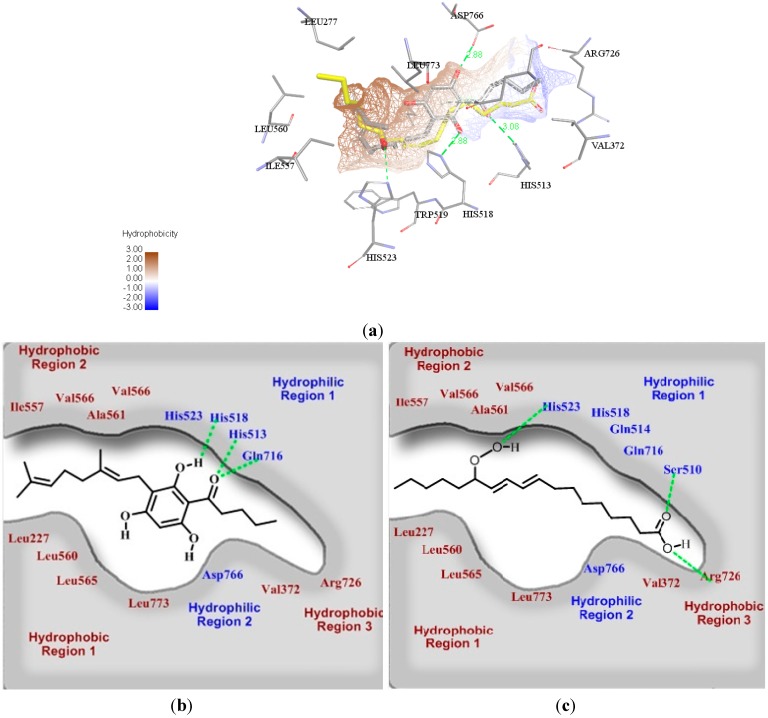
(**a**) Three-dimensional (3-D) docking model of the superimposed structures of the most active compound **3e** with the (9*Z*,11*E*)-13(*R*)-hydroperoxy-9,11-octadecadienoic acid (13-HPOD) as LOX substrate. The grey color represents compound **3e**, while yellow sticks represents 13-HPOD, with distance indicated in angstroms (Å);(**b**) Two-dimensional (2-D) diagram of binding interaction between the most active compound **3e** with amino acid residues of soybean 15-LOX; (**c**) Two-dimensional (2-D) diagram of binding interaction between 13-HPOD with amino acid residues of soybean 15-LOX. The green color line indicates the hydrogen bonding.

To further understand the binding conformation of **3e** in the LOX active site, we first retrieved the docked conformation of **3e** and then superimposed it with the original co-crystalized ligand, (9*Z*,11*E*)-13(*R*)-hydroperoxy-9,11-octadecadienoic acid (13-HPOD), found in the ligand-protein complex crystal structure. Figure 5a and Figure 5b show that compound **3e** binds in the similar cavity as 13-HPOD (Figure 5c). Compound **3g** was also predicted to occupy the same cavity of 13-HPOD (Supplementary data Figure S37). Interestingly, from our previous study, tHGA was found to be a non-redox competitive inhibitor where there is no complexation between tHGA and the metal ions (Fe^2+^, Fe^3+^ and Cu^2+^) [17]. Thus, it is highly suggested that the inhibition of soybean 15-LOX activity by compound **3e** could be due to the competition with 13-HPOD to occupy the active site.

Once compound **3e** successfully binds to the catalytic region, it could possibly distort the conformation of His518 residue which is involved in the formation or stabilization of radical(s) via proton and/or electron transfer [28]. Closer inspection shows that the imidazole side chain of histidine residue interacts with the hydroxyl substituent of **3e** through hydrogen bonding interaction in the hydrophilic region 1 as depicted in Figure 5b. This specific interaction was not observed for NDGA (Supplementary data Figure S38) retrieved from the docking results. We suspect that NDGA and compound **3e** each bind in a different manner in the LOX active site. Lastly, the geranyl group of **3e** is well positioned in the hydrophobic region 1 and 2, constituting of hydrophobic residues Leu227, Leu560, Leu565, Leu773, Ile557, Val566 and Ala561. The presence of the geranyl group could possibly increase the ligand binding affinity of **3e** through hydrophobic interactions. Besides, the longer aliphatic chain on the acyl moiety of **3e** allows the penetration into the hydrophobic region 3, constituting of hydrophobic Val372 and Asp726 residues, respectively (Figure 5b), and thus resulted in higher inhibitory activity of **3e**.

## 3. Experimental Section

### 3.1. Synthesis of 2,4,6-Trihydroxy-3-Geranylacetophenone (tHGA) Analogs

#### 3.1.1. General Methods

All reagents and solvents used were reagent grade. Aluminum sheets precoated with Silica Gel 60 F254 (20 × 20 cm, 0.2 mm thick; Merck) were used for TLC and silica gel 60 (0.040–0.063 mm) and silica gel 60 (0.063–0.200 mm) (Merck) was used for column chromatography. NMR and mass analysis were carried out using 500 MHz Varian and DIMS QP5050A SHIMADZU, respectively.

#### 3.1.2. Friedel-Craft Acylation

The mixture of phloroglucinol (0.05 mol) and anhydrous aluminum chloride (0.1 mol) were dissolved in dichloromethane (50 mL). Phloroglucinol and aluminum chloride easily dissolved in the solvent and HCl was observed to be produced when the reaction temperature rose to about 33 °C. The mixture was heated for 10 min at 40 °C and the acyl chloride (0.05 mol) was added dropwise. After refluxing for 15 min, 0.05 mol of HCl was added to quench the reaction. The solvent was evaporated off, and the resultant product extracted with ethyl acetate. The ethyl acetate extract was purified using flash column chromatography over silica gel, eluted with petroleum ether/ethyl acetate (10:1) [22].

*2-Methyl-1-(2,4,6-trihydroxyphenyl)propan-1-one* (**2a**). Light yellow solid, yield 6.75%. ^1^H-NMR (500 MHz, Methanol-d_4_) d 5.81 (s, 2H), 3.97 (td, *J* = 6.82, 13.51 Hz, 1H), 1.13 (d, *J =* 6.60 Hz, 6H). ^13^C-NMR (126 MHz, Methanol-d_4_) 210.3, 164.2, 164.0, 103.2, 94.5, 38.5, 18.3. DIP: *m/z* 196.05. 

*1-(2,4,6-Trihydroxyphenyl)propan-1-one* (**2c**). Light brown solid, yield 2.81%. ^1^H-NMR (500 MHz, Methanol-d_4_) d 5.80 (s, 2H), 3.04–3.07 (m, 2H), 1.12 (dt, *J* = 1.47, 7.21 Hz, 3H). ^13^C-NMR (126 MHz, Methanol-d_4_) 206.5, 164.5, 164.3, 103.8, 94.4, 36.5, 7.8. DIP: *m/z* 182.05.

*2,2-Dimethyl-1-(2,4,6-trihydroxyphenyl)propane-1-one* (**2d**). Dark brown solid, yield 0.75%. ^1^H-NMR (500 MHz, Methanol-d_4_) d 5.83 (s, 2H), 1.21 (s, 9H). ^13^C-NMR (126 MHz, Methanol-d_4_) 216.4, 159.0, 155.6, 109.5, 93.9, 44.6, 26.1. DIP: *m/z* 210.05.

*1-(2,4,6-Trihydroxyphenyl)pentan-1-*one (**2e**). Light yellow solid, yield 12.5%. ^1^H-NMR (500 MHz, Methanol-d_4_) d 5.80 (s, 2H), 3.03 (t, *J* = 7.46 Hz, 2H), 1.63 (quin, *J* = 7.46 Hz, 2H), 1.38 (sxt, *J* = 7.39 Hz, 2H), 0.94 (t, 3H). ^13^C-NMR (126 MHz, Methanol-d_4_) 206.2, 164.5, 164.4, 104.0, 94.4, 43.1, 27.0, 22.3, 12.9. DIP: *m/z* 210.10. 

*Cyclohexyl(2,4,6-trihydroxyphenyl)methanone* (**2f**). Orange brown solid, yield 14.15%. ^1^H-NMR (500 MHz, Methanol-d_4_) d 5.81 (s, 2H), 3.67–3.71 (m, 1H), 1.89 (br. s., 2H), 1.78 (dd, *J* = 2.69, 9.05 Hz, 2H), 1.69 (d, *J* = 12.47 Hz, 1H), 1.30–1.41 (m, 5H), 1.21–1.25 (m, 1H). ^13^C-NMR (126 MHz, Methanol-d_4_) 209.40, 165.4, 164.30, 103.44, 94.54, 49.19 , 29.31, 25.94, 25.90. DIP: *m/z* 236.10. 

*(E)-3-Phenyl-1-(2,4,6-trihydroxyphenyl)prop-2-en-1-one* (**2g**). Orange solid, yield 9.1%. ^1^H-NMR (500 MHz, Methanol-d_4_) d 8.23 (d, *J* = 15.65 Hz, 1H), 7.73 (d, *J* = 15.65 Hz, 1H), 7.62 (d, *J* = 6.60 Hz, 2H), 7.37–7.42 (m, 4H), 5.86 (s, 2H). ^13^C-NMR (126 MHz, Methanol-d_4__4_) 192.6, 165.2, 164.7, 141.4, 135.7, 129.6, 128.5, 128.0, 127.9, 127.5, 104.5, 94.6. DIP: *m/z* 256.05.

#### 3.1.3. Friedel-Craft Alkylation

A well-mixed mixture of phloracetophenone (1 mmol), geranyl bromide (1 mmol), and anhydrous potassium carbonate (0.5 mmol) in dry methanol (3 mL) was refluxed for 8 h. The resultant products were extracted with ethyl acetate. The organic layers were combined, dried and concentrated. The extract was redissolved in ethyl acetate and purified using flash column chromatography over silica gel, eluted with petroleum ether/ethyl acetate (10:1) [18].

*3-Geranyl-1-(2'-methylpropanoyl)phloroglucinol* (**3a**). Orange brown solid, yield 19.5%.^ 1^H-NMR (500 MHz, Methanol-d_4_) d 5.88 (s, 1H), 5.13–5.19 (m, 1H), 5.00–5.08 (m, 1H), 3.94–4.03 (m, 1H), 3.17 (br. s., 2H), 2.03 (br. s., 2H), 1.93 (br. s., 2H), 1.73 (s, 3H), 1.59 (br. s., 3H), 1.54 (s, 3H), 1.12 (dd, *J =* 3.64, 6.55 Hz, 6H). ^13^C-NMR (126 MHz, Methanol-d_4_) 211.8, 165.4, 163.5, 161.1, 134.7, 132.0, 125.6, 124.7, 108.2, 104.6, 95.0, 41.0, 39.9, 27.8, 25.9, 22.2, 19.8, 17.7, 16.2. DIP: *m/z* 332.10.

*(E)-1-(3-(3,7-Dimethylocta-2,6-dienyl)-2,4,6-trihydroxyphenyl)propan-1-one* (**3c**). Yellowish brown solid, yield 6.9%. ^1^H-NMR (500 MHz, Methanol-d_4_) d 5.89 (s, 1H), 5.16–5.23 (m, 1H), 5.06–5.10 (m, 1H), 3.17 (d, *J =* 7.09 Hz, 2H), 3.04-3.07 (m, 2H), 1.95–2.05 (m, 2H), 1.92 (d, *J =* 7.83 Hz, 2H), 1.73 (s, 3H), 1.60 (s, 3H), 1.55 (s, 3H), 1.13 (t, *J =* 7.21 Hz, 3H). ^13^C-NMR (126 MHz, Methanol-d_4_) 206.5, 163.4, 162.1, 160.0, 133.3, 130.5, 124.1, 123.2, 106.6, 103.7, 93.4, 39.5, 36.6, 26.3, 24.4, 20.7, 16.3, 14.8, 8.0. DIP: *m/z* 318.20.

*(E)-1-(3-(3,7-Dimethylocta-2,6-dienyl)-2,4,6-trihydroxyphenyl)pentan-1-one* (**3e**). Yellowish brown solid, yield 10.3%. ^1^H-NMR (500 MHz, Methanol-d_4_) d 5.90 (s, 1H), 5.13–5.19 (m, 1H), 5.04 (br. s., 1H), 3.17 (d, *J =* 6.85 Hz, 2H), 3.03 (t, *J =* 1.00 Hz, 2H), 2.03–2.04 (m, 2H), 1.91–1.94 (m, 2H), 1.73 (s, 3H), 1.61–1.67 (m, 2H), 1.60 (s, 3H), 1.54 (s, 3H), 1.38–1.41 (m, 2H), 0.94 (t, *J =* 7.34 Hz, 3H).^ 13^C-NMR (126 MHz, Methanol-d_4_) 206.3, 163.5, 156.8, 133.3, 130.6, 124.1, 123.2, 43.2, 39.5, 27.2, 26.3, 24.4, 22.3, 20.7, 16.3, 14.8, 12.9. DIP: *m/z* 346.20. 

*(E)-Cyclohexyl (3-(3,7-dimethylocta-2,6-dienyl)-2,4,6-trihydroxyphenyl)-methanone* (**3f**). Brown solid, yield 5.7%. ^1^H-NMR (500 MHz, Methanol-d_4_) d 5.89 (s, 1H), 5.14–5.20 (m, 1H), 5.02–5.08 (m, 1H), 3.68–3.76 (m, 1H), 3.17 (d, *J =* 7.09 Hz, 2H), 2.04 (d, *J =* 7.34 Hz, 2H), 1.86–1.97 (m, 4H), 1.80 (d, *J* = 11.00 Hz, 2H), 1.73 (s, 3H), 1.60 (s, 3H), 1.55 (s, 3H), 1.31–1.44 (m, 4H), 1.23–1.30 (m, 2H). ^13^C-NMR (126 MHz, Methanol-d_4_) 209.4, 163.9, 161.9, 159.6, 133.2, 130.5, 124.1, 123.3, 106.8, 103.2, 93.6, 49.2, 39.5, 29.4, 26.3, 26.0, 25.9, 24.4, 20.7, 16.3, 14.8. DIP: *m/z* 372.05.

*(E)-1-(3-((E)-3,7-Dimethylocta-2,6-dienyl)-2,4,6-trihydroxyphenyl)-3-phenylprop-2-en-1-one* (**3g**). Light yellow solid, yield 11.7%. ^1^H-NMR (500 MHz, Methanol-d_4_) d 7.48–7.50 (m, 2H), 7.39–7.43 (m, 2H), 7.34–7.38 (m, 1H), 5.97 (s, 1H), 5.17–5.22 (m, 1H), 5.04–5.08 (m, 1H), 3.23 (d, *J =* 1.00 Hz, 2H), 3.03–3.11 (m, 1H), 2.73–2.79 (m, 1H), 2.02-2.08 (m, 2H), 1.92–1.98 (m, 2H), 1.75 (s, 3H), 1.62 (s, 3H), 1.56 (s, 3H). ^13^C-NMR (126 MHz, Methanol-d_4_) 195.8, 165.2, 161.1, 160.9, 139.2, 133.8, 130.6, 128.4, 128.2, 128.1, 125.9, 124.1, 122.6, 108.5, 101.7, 94.3, 78.9, 42.9, 39.5, 26.3, 24.4, 20.4, 16.3, 14.8. DIP: *m/z* 392.20.

### 3.2. In Vitro Soybean 15-Lipoxygenase (LOX) Inhibition Assay

*In vitro* soybean 15-LOX inhibiting activity was measured using spectrophotometric method. In brief, sodium phosphate buffer (160 μL, 100 mM, pH 8.0), test sample (10 μL) and soybean 15-LOX (1.13.11.12) type I-B solution (20 μL, 320 U/well) were mixed and incubated for 15 min at 25 °C. The reaction was then initiated by the addition of the substrate in the form of linoleic acid (10 μL, 0.6 mM) solution. The enzymatic conversion of linoleic acid to form (9*Z*,11*E*)-(13*S*))-13-hydroperoxyoctadeca-9,11-dienoate end product was followed by the change of absorbance measured at 234 nm over a period for 6 min. Test samples and reference standards were dissolved in DMSO. Stock solutions were prepared at concentration 100 μg/mL for pure compounds. Pure compounds were tested at a final concentration of 100 μg/mL and for compound showing bioactivity, the IC_50_ value was determined from tests concentrations of 100, 50, 25, 12.5 6.25, 3.13 and 1.57 μg/mL. For reference compound Nordihydroguaiaretic acid (NDGA), the IC_50_ value was determined from tests concentrations of 25, 12.5, 6.25, 3.13, 1.57, 0.79, 0.4, 0.2, 0.1, 0.05, 0.025, 0.0125 and 0.00625 μg/mL. All reactions were performed in triplicate in a 96-well microtitre plate. The IC_50_ was calculated from the non-linear regression fitting curve of GraphPad Prism 5. The percentage of inhibition (%) can be calculated from the following formula [17]:

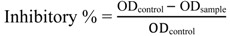
(1)


### 3.3. Molecular Docking

Docking studies were performed with the cDOCKER protocol under the receptor-ligand interaction section in Discovery Studio^®^ 3.1 (Accelrys, Inc., San Diego, CA, USA). All of the 3D structures of the compounds were built with ChemBioOffice^®^ 2008 (PerkinElmer, Inc., Waltham, MA, USA). The protein crystal structure of the inhibitor-bound soybean 15-LOX was retrieved from the Brookhaven Protein Data Bank (PDB Code: **1IK3**: 2.0 Å). Protein was pre-treated before the docking. Hydrogen atoms were added to the protein structure, and all ionisable residues were set at their default protonation of pH 7.4 while the ligands were prepared and minimized. During the docking process, the receptor was held rigid while the ligands were allowed to flex during the refinement. The ligands were heated to 700 K in 2000 steps, and the cooling temperature was set to 300 K in 5000 steps. Grid extension was set to 10 Å. Finally, 10 ligand-binding poses were ranked according to their cDOCKER energy, and the predicted binding interactions were analyzed [29].

## 4. Conclusions

In conclusion, a series of 2,4,6-trihydroxy-3-geranyl-acetophenone (tHGA) analogs have been successfully synthesized. Biological evaluation revealed that the three target compounds (**3c**, **3e** and **3g**) displayed better activities against soybean15-LOX with IC_50 _ values 12.32 μM, 10.32 μM and 15.20 μM, respectively. The elongation of aliphatic chain of acyl moieties (**3c** and **3e**) and introduction of aromatic moiety (**3g**) significantly improved the inhibitory activity as compared to tHGA (IC_50_ = 23.61 μM), respectively. On the other hand, molecular docking studies revealed that the most active compound **3e** was found to line in the same cavity of 13-HPOD. Thus, it is suggested that the inhibition of soybean15-LOX could be through competitive inhibition. Therefore, our findings support that these geranylacetophenones has promising potential as lead compounds for the design of new anti-inflammatory drugs or NSAIDs. The significant improvement in bioactivities of these analogs are worthy of further pharmacological evaluation and optimization as potential LOX-inhibitors.

## Supplementary Files

Supplementary File 1

## Supplementary Materials

Supplementary materials can be accessed at: http://www.mdpi.com/1420-3049/19/8/11645/s1.
