# Supplementary material for: Synthesis and Docking Studies of 2,4,6-Trihydroxy-3-Geranylacetophenone Analogs as Potential Lipoxygenase Inhibitor
Source: Molecules. 2014 Aug 5;19(8):11645–59. doi: 10.3390/molecules190811645 (PMC6271415; doi:10.3390/molecules190811645)

Supplementary Materials

Figure S1. Chemical structures of phloracetophenone intermediates (**2a**, **2c**–**2g**).

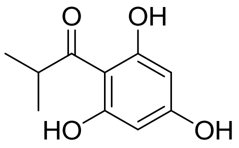

2a

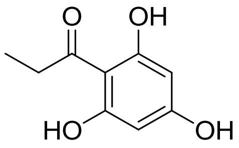

2c

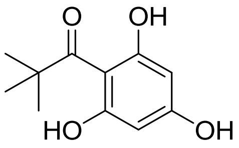

2d

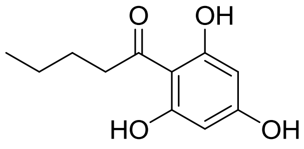

2e

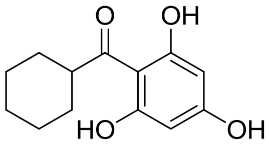

2f

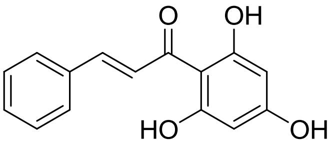

2g

Figure S2. Chemical structures of geranylacetophenone analogues (**3a**, **3c**, **3e**–**g**).

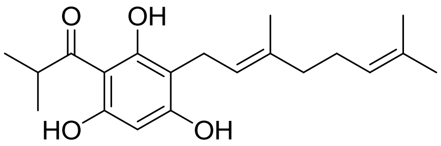

3a

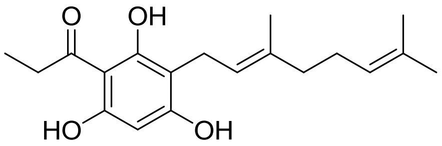

3c

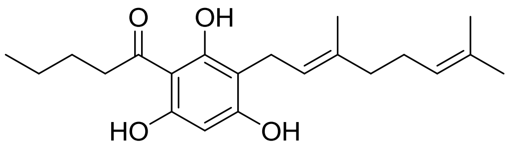

3e

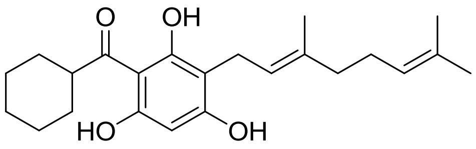

3f

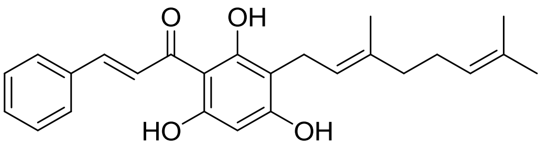

3g

2-Methyl-1-(2,4,6-trihydroxyphenyl)propan-1-one (**2a**). Light yellow solid, yield 6.75%. ^{1}H-NMR (500 MHz, Methanol-d_4) d 5.81 (s, 2H), 3.97 (td, J = 6.82, 13.51 Hz, 1H), 1.13 (d, J = 6.60 Hz, 6H). ^{13}C-NMR (126 MHz, Methanol-d_4) 210.3, 164.2, 164.0, 103.2, 94.5, 38.5, 18.3. DIP: *m/z* 196.05.

Figure S3. ^{1}H-NMR spectrum of 2-methyl-1-(2,4,6-trihydroxyphenyl)propan-1-one.

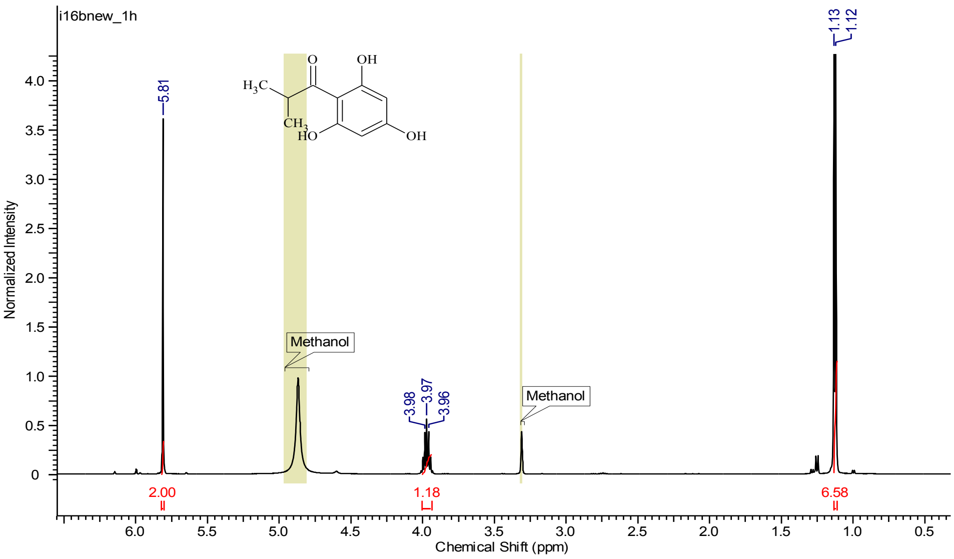

Figure S4. ^{13}C-NMR spectrum of 2-methyl-1-(2,4,6-trihydroxyphenyl)propan-1-one.

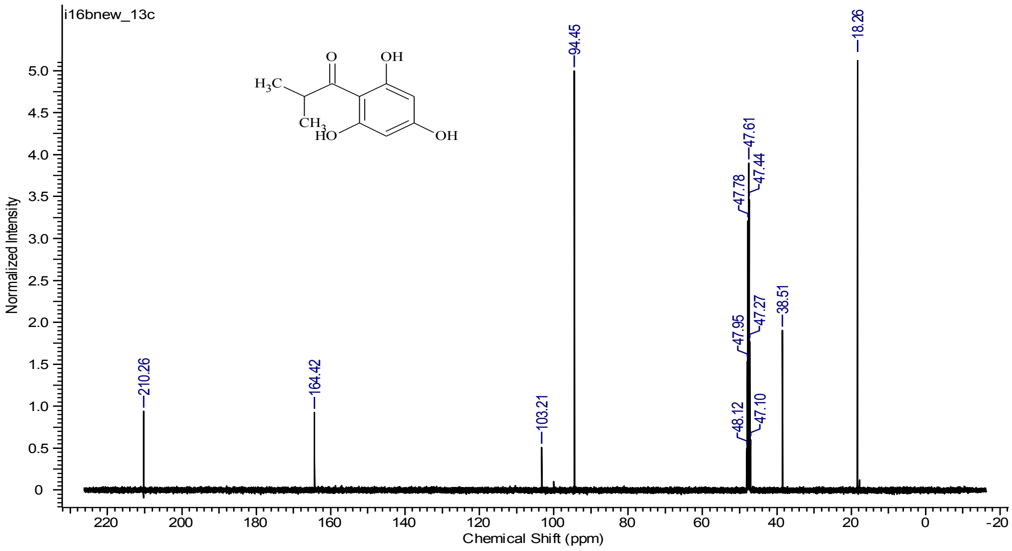

Figure S5. Mass spectrum of 2-methyl-1-(2,4,6-trihydroxyphenyl)propan-1-one.

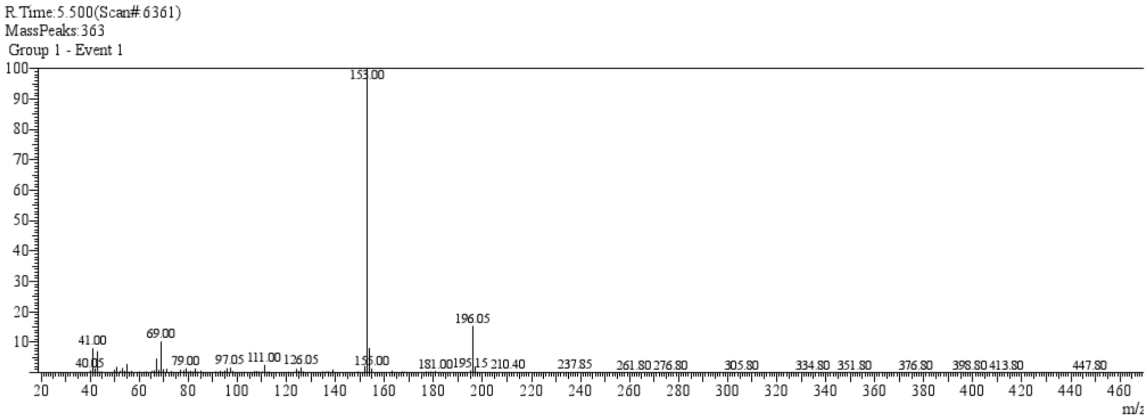

1-(2,4,6-Trihydroxyphenyl)propan-1-one (**2c**). Light brown solid, yield 2.81%. ^{1}H-NMR (500 MHz, Methanol-d_4) d 5.80 (s, 2H), 3.04–3.07 (m, 2H), 1.12 (dt, J = 1.47, 7.21 Hz, 3H). ^{13}C-NMR (126 MHz, Methanol-d_4) 206.5, 164.5, 164.3, 103.8, 94.4, 36.5, 7.8. DIP: m/z 182.05.

Figure S6. ^{1}H-NMR spectrum of 1-(2,4,6-trihydroxyphenyl)propan-1-one.

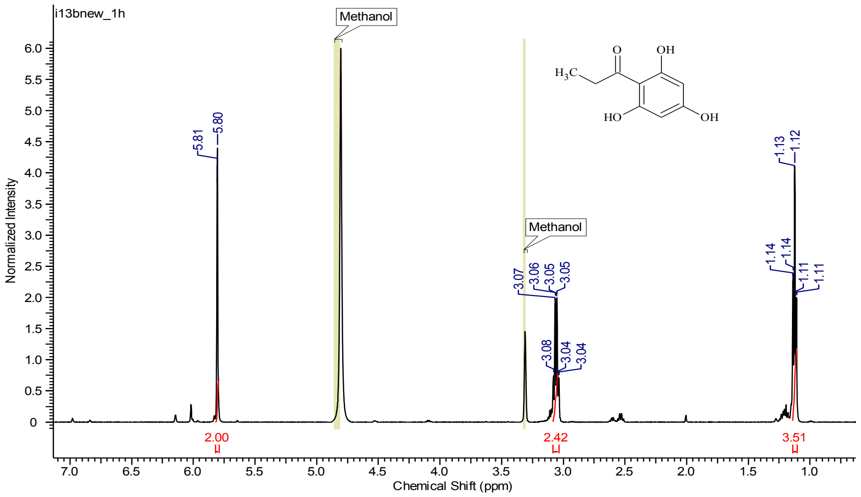

Figure S7. ^{13}C-NMR spectrum of 1-(2,4,6-trihydroxyphenyl)propan-1-one.

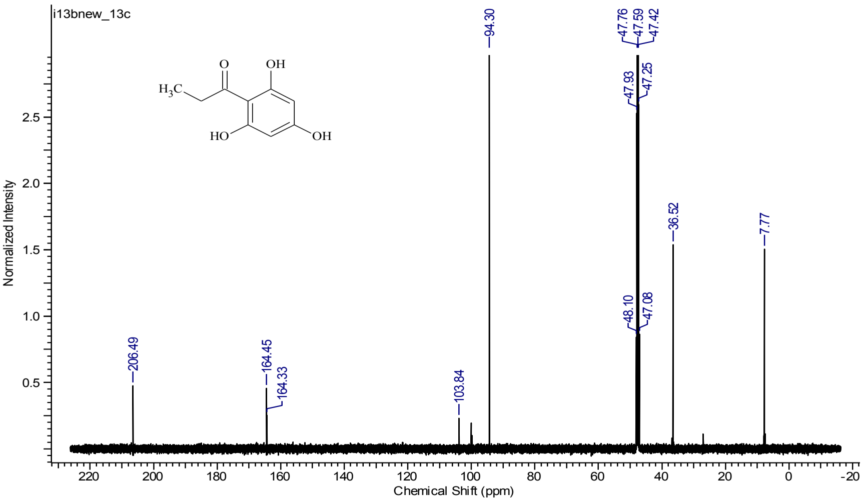

Figure S8. Mass spectum of 1-(2,4,6-trihydroxyphenyl)propan-1-one.

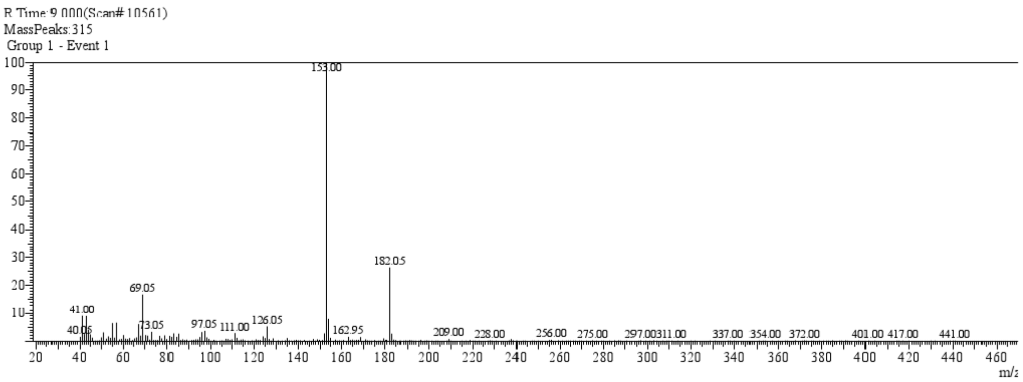

2,2-Dimethyl-1-(2,4,6-trihydroxyphenyl)propane-1-one (**2d**). Dark brown solid, yield 0.75%. ^{1}H-NMR (500 MHz, Methanol-d$_4$) d 5.83 (s, 2H), 1.21 (s, 9H). ^{13}C-NMR (126 MHz, Methanol-d$_4$) 216.4, 159.0, 155.6, 109.5, 93.9, 44.6, 26.1. DIP: *m/z* 210.05.

Figure S9. ^{1}H-NMR spectrum of 2,2-dimethyl-1-(2,4,6-trihydroxyphenyl)-propane-1-one.

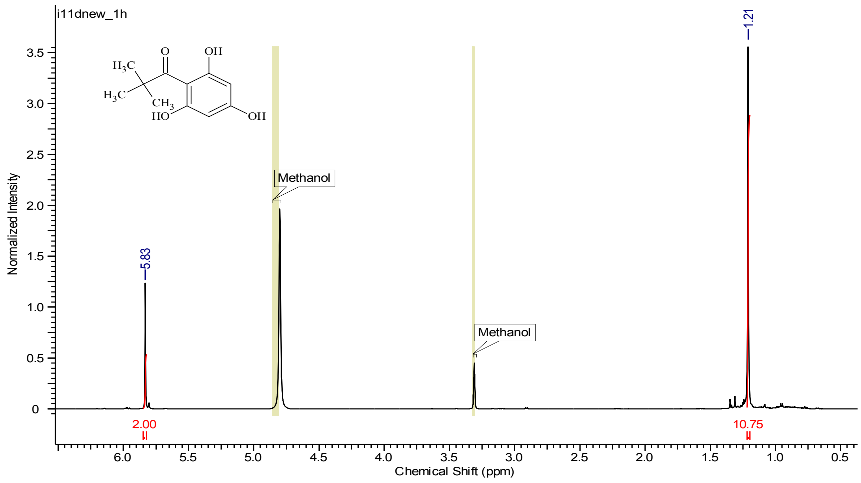

Figure S10. ^{13}C-NMR spectrum of 2,2-dimethyl-1-(2,4,6-trihydroxyphenyl)-propane-1-one.

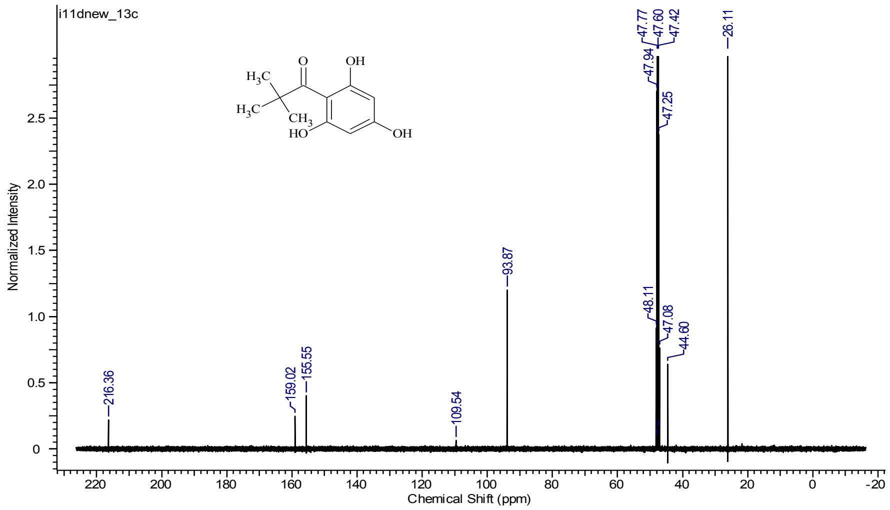

Figure S11. Mass spectrum of 2,2-dimethyl-1-(2,4,6-trihydroxyphenyl)-propane-1-one.

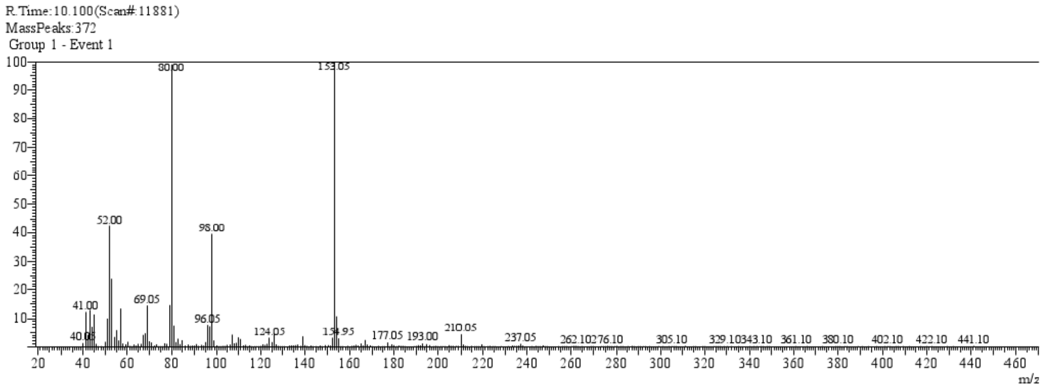

1-(2,4,6-Trihydroxyphenyl)pentan-1-one (**2e**). Light yellow solid, yield 12.5%. ^{1}H-NMR (500 MHz, Methanol-d_4) d 5.80 (s, 2H), 3.03 (t, J = 7.46 Hz, 2H), 1.63 (quin, J = 7.46 Hz, 2H), 1.38 (sxt, J = 7.39 Hz, 2H), 0.94 (t, 3H). ^{13}C-NMR (126 MHz, Methanol-d_4) 206.2, 164.5, 164.4, 104.0, 94.4, 43.1, 27.0, 22.3, 12.9. DIP: *m/z* 210.10.

Figure S12. ^{1}H-NMR spectrum of 1-(2,4,6-trihydroxyphenyl)pentan-1-one.

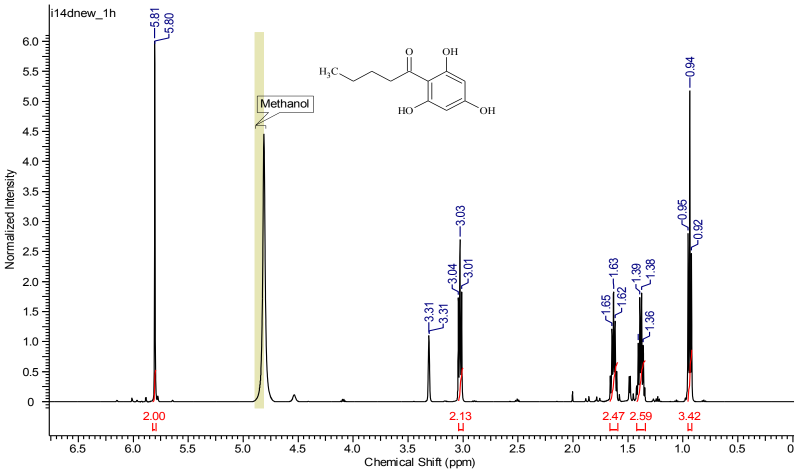

Figure S13. ^{13}C-NMR spectrum of 1-(2,4,6-trihydroxyphenyl)pentan-1-one.

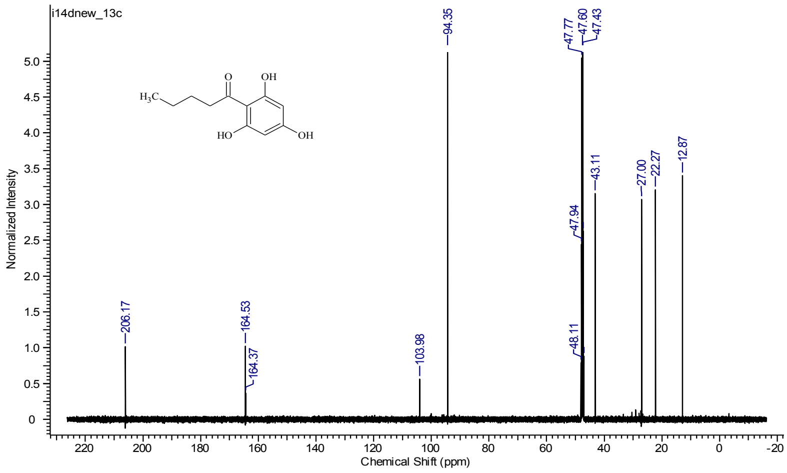

Figure S14. Mass spectrum of 1-(2,4,6-trihydroxyphenyl)pentan-1-one.

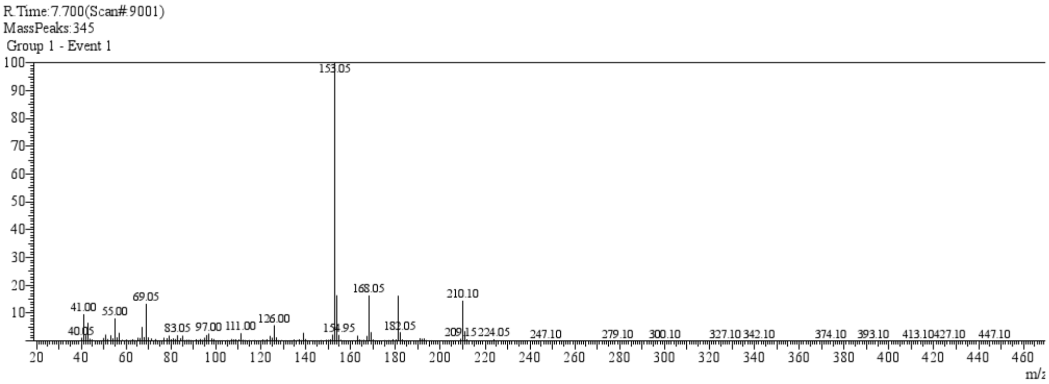

Cyclohexyl(2,4,6-trihydroxyphenyl)methanone (**2f**). Orange brown solid, yield 14.15%. ^{1}H-NMR (500 MHz, Methanol-d_4) d 5.81 (s, 2H), 3.67–3.71 (m, 1H), 1.89 (br. s., 2H), 1.78 (dd, J = 2.69, 9.05 Hz, 2H), 1.69 (d, J = 12.47 Hz, 1H), 1.30–1.41 (m, 5H), 1.21–1.25 (m, 1H). ^{13}C-NMR (126 MHz, Methanol-d_4) 209.40, 165.4, 164.30, 103.44, 94.54, 49.19 , 29.31, 25.94, 25.90. DIP: m/z 236.10.

Figure S15. ^{1}H-NMR spectrum of cyclohexyl(2,4,6-trihydroxyphenyl)methanone.

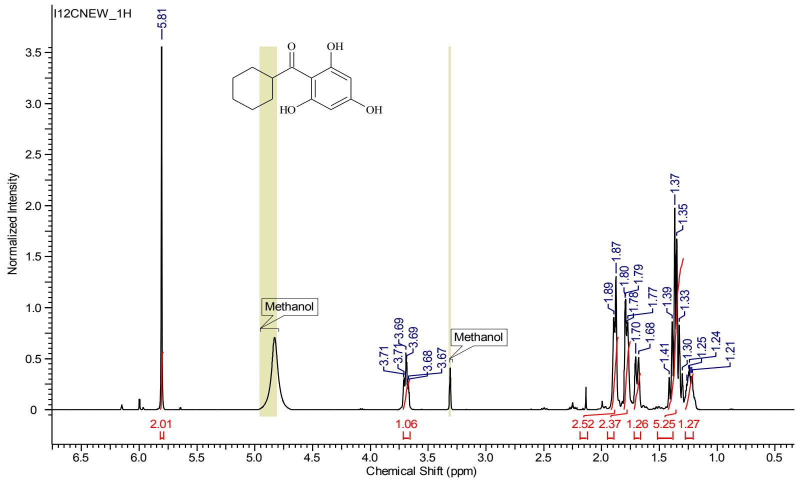

Figure S16. ^{13}C-NMR spectrum of cyclohexyl(2,4,6-trihydroxyphenyl)methanone.

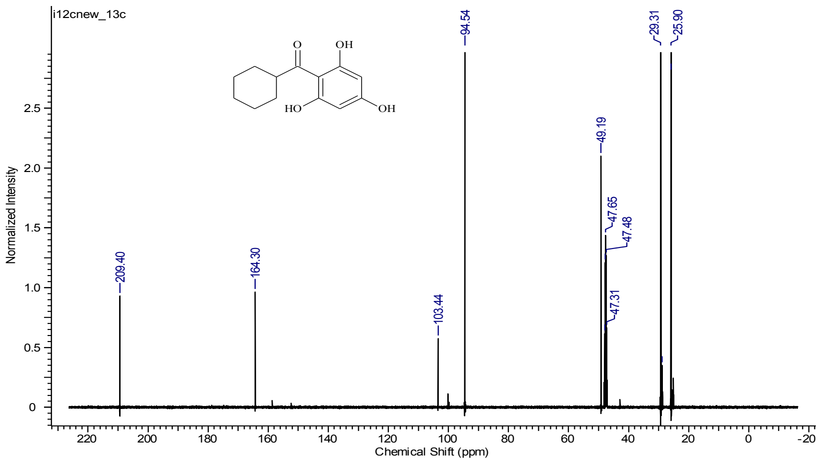

Figure S17. Mass spectrum of cyclohexyl(2,4,6-trihydroxyphenyl)methanone.

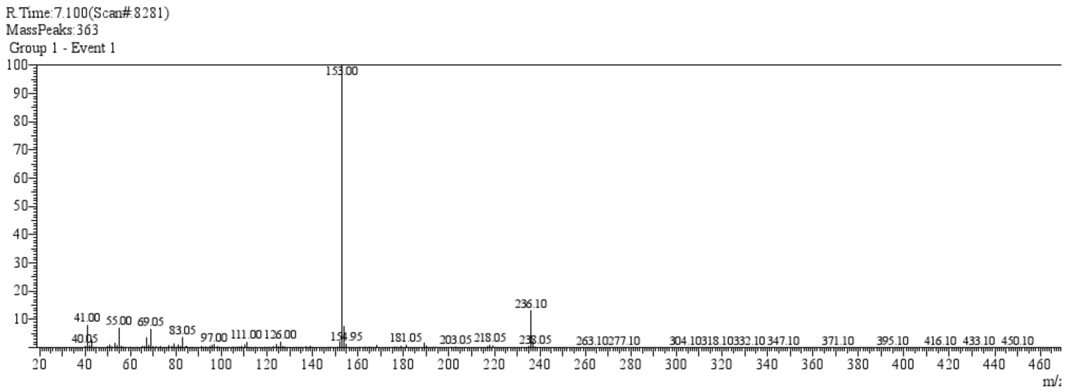

(E)-3-Phenyl-1-(2,4,6-trihydroxyphenyl)prop-2-en-1-one (**2g**). Orange solid, yield 9.1%. ^{1}H-NMR (500 MHz, Methanol-d_4) d 8.23 (d, *J* = 15.65 Hz, 1H), 7.73 (d, *J* = 15.65 Hz, 1H), 7.62 (d, *J* = 6.60 Hz, 2H), 7.37–7.42 (m, 4H), 5.86 (s, 2H). ^{13}C-NMR (126 MHz, Methanol-d_{44}) 192.6, 165.2, 164.7, 141.4, 135.7, 129.6, 128.5, 128.0, 127.9, 127.5, 104.5, 94.6. DIP: *m/z* 256.05.

Figure S18. ^{1}H-NMR spectrum of (*E*)-3-phenyl-1-(2,4,6-trihydroxyphenyl)- prop-2-en-1-one.

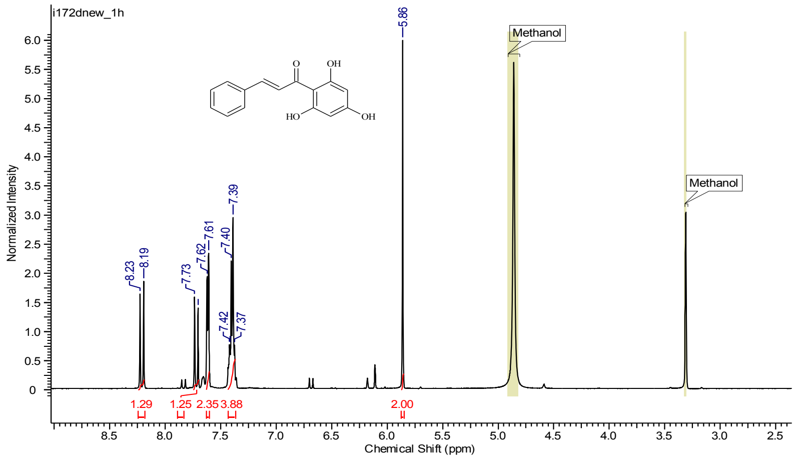

Figure S19. ^{13}C-NMR spectrum of (*E*)-3-phenyl-1-(2,4,6-trihydroxyphenyl)- prop-2-en-1-one.

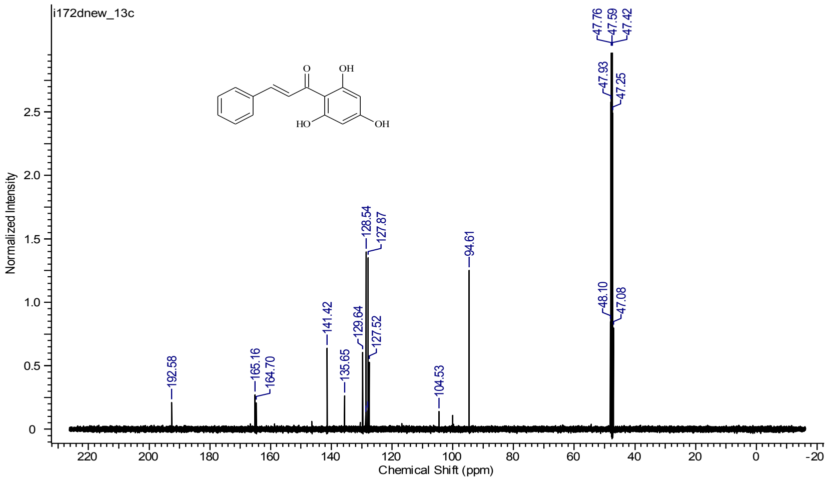

Figure S20. Mass spectrum of (*E*)-3-phenyl-1-(2,4,6-trihydroxyphenyl)- prop-2-en-1-one.

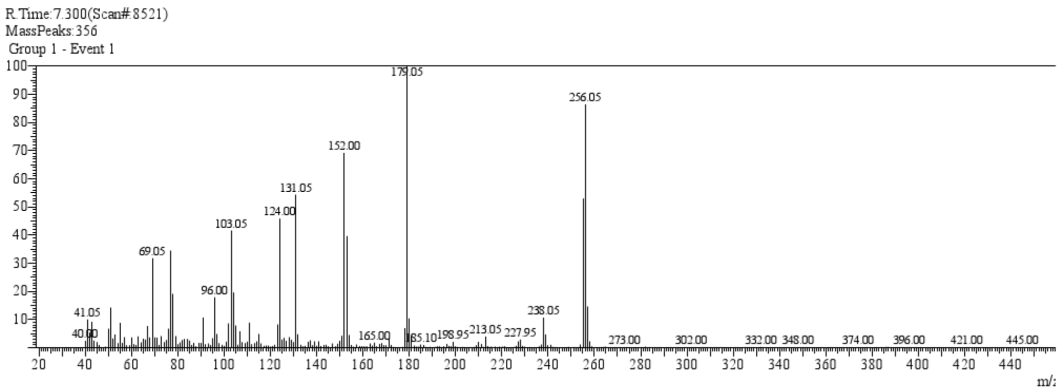

3-Geranyl-1-(2'-methylpropanoyl)phloroglucinol (**3a**). Orange brown solid, yield 19.5%. ^{1}H-NMR (500 MHz, Methanol-d_4) d 5.88 (s, 1H), 5.13–5.19 (m, 1H), 5.00–5.08 (m, 1H), 3.94–4.03 (m, 1H), 3.17 (br. s., 2H), 2.03 (br. s., 2H), 1.93 (br. s., 2H), 1.73 (s, 3H), 1.59 (br. s., 3H), 1.54 (s, 3H), 1.12 (dd, J = 3.64, 6.55 Hz, 6H). ^{13}C-NMR (126 MHz, Methanol-d_4) 211.8, 165.4, 163.5, 161.1, 134.7, 132.0, 125.6, 124.7, 108.2, 104.6, 95.0, 41.0, 39.9, 27.8, 25.9, 22.2, 19.8, 17.7, 16.2. DIP: m/z 332.10.

Figure S21. ^{1}H-NMR spectrum of 3-geranyl-1-(2'-methylpropanoyl)phloroglucinol.

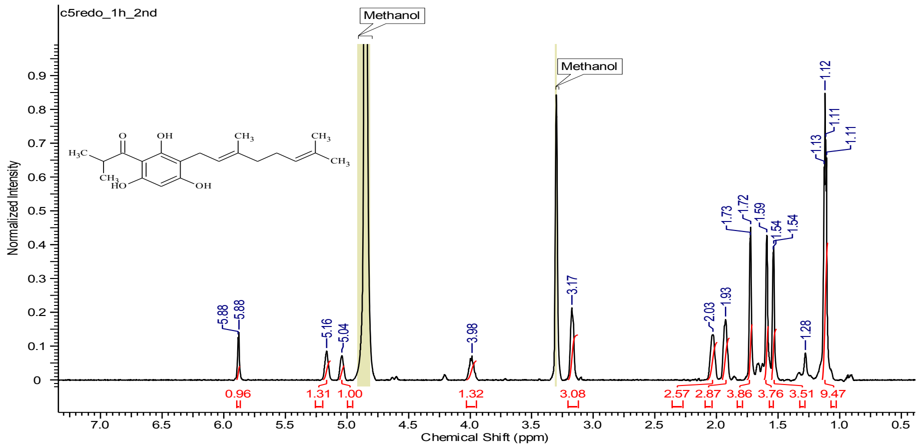

Figure S22. ^{13}C-NMR spectrum of 3-geranyl-1-(2'-methylpropanoyl)phloroglucinol.

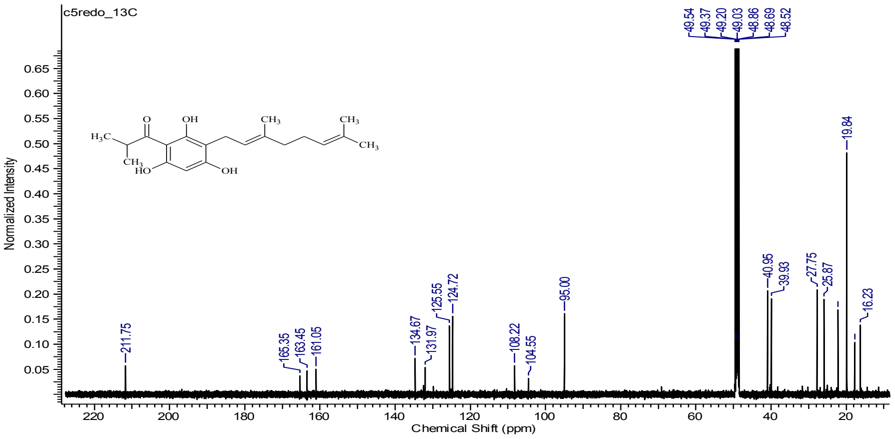

Figure S23. Mass spectrum of 3-geranyl-1-(2'-methylpropanoyl)phloroglucinol.

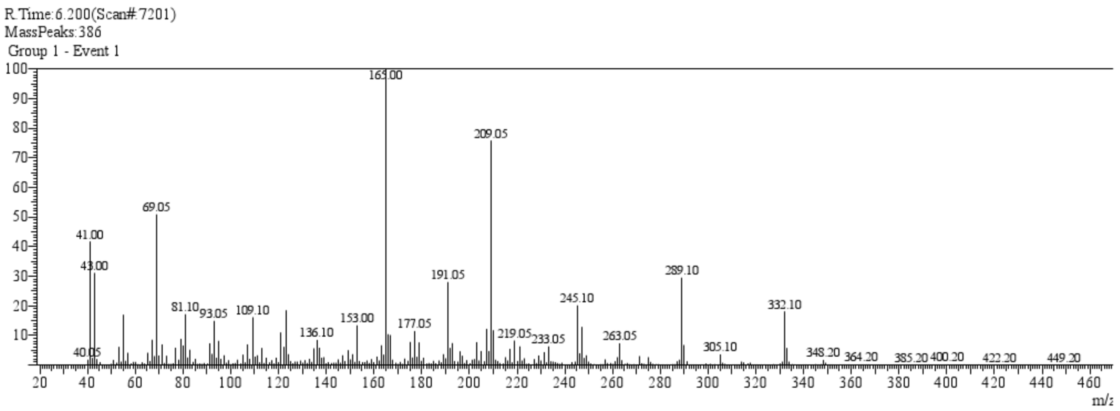

(E)-1-(3-(3,7-Dimethylocta-2,6-dienyl)-2,4,6-trihydroxyphenyl)propan-1-one (**3c**). Yellowish brown solid, yield 6.9%. ^{1}H-NMR (500 MHz, Methanol-d$_4$) d 5.89 (s, 1H), 5.16–5.23 (m, 1H), 5.06–5.10 (m, 1H), 3.17 (d, J = 7.09 Hz, 2H), 3.04-3.07 (m, 2H), 1.95–2.05 (m, 2H), 1.92 (d, J = 7.83 Hz, 2H), 1.73 (s, 3H), 1.60 (s, 3H), 1.55 (s, 3H), 1.13 (t, J = 7.21 Hz, 3H). ^{13}C-NMR (126 MHz, Methanol-d$_4$) 206.5, 163.4, 162.1, 160.0, 133.3, 130.5, 124.1, 123.2, 106.6, 103.7, 93.4, 39.5, 36.6, 26.3, 24.4, 20.7, 16.3, 14.8, 8.0. DIP: m/z 318.20.

Figure S24. ^{1}H-NMR spectrum of (*E*)-1-(3-(3,7-dimethylocta-2,6-dienyl)-2,4,6-trihydroxyphenyl)-propan-1-one.

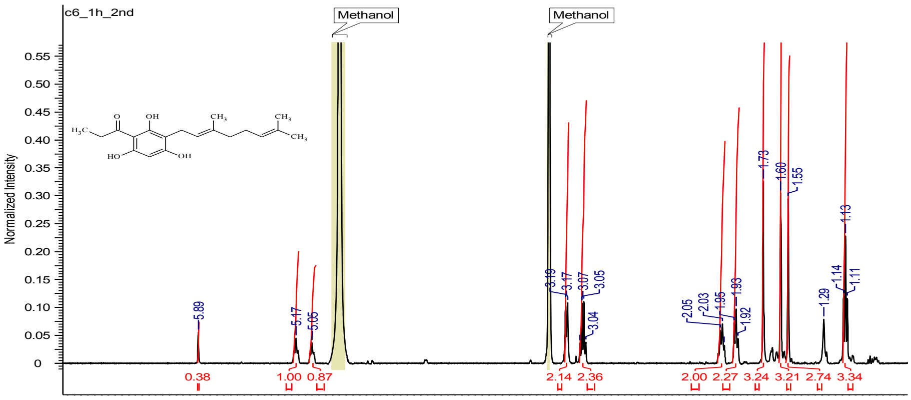

Figure S25. ^{13}C-NMR spectrum of (*E*)-1-(3-(3,7-dimethylocta-2,6-dienyl)-2,4,6-trihydroxyphenyl)-propan-1-one.

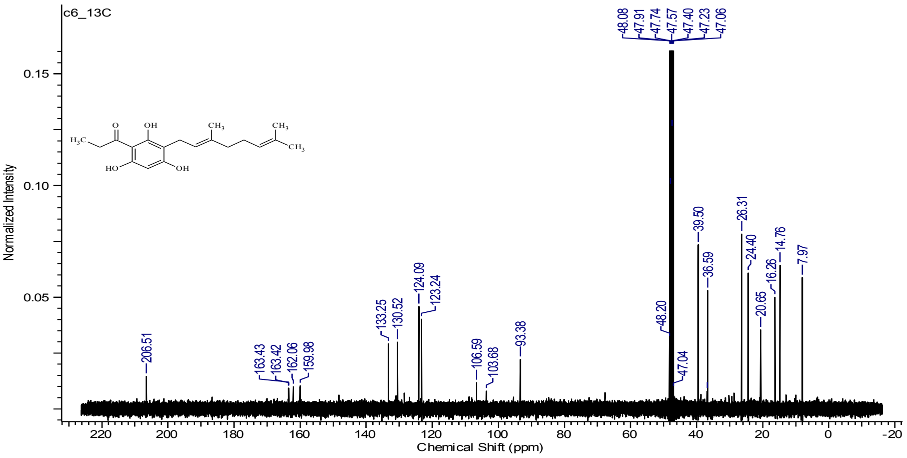

Figure S26. Mass spectrum of (*E*)-1-(3-(3,7-dimethylocta-2,6-dienyl)-2,4,6-trihydroxyphenyl)-propan-1-one.

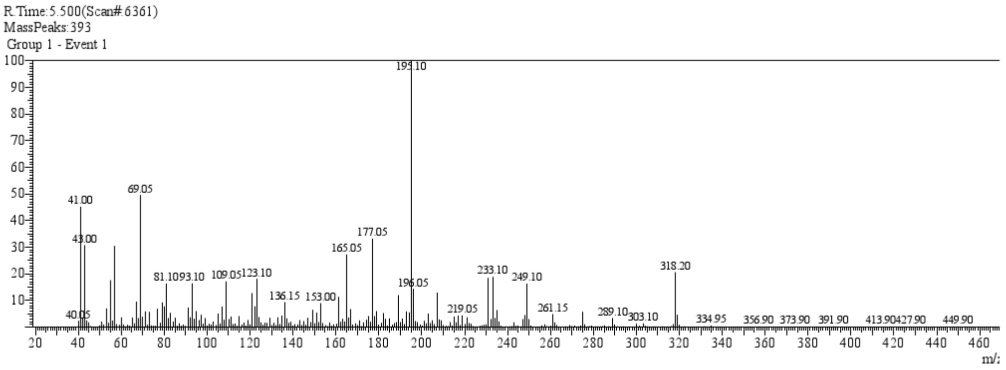

(E)-1-(3-(3,7-Dimethylocta-2,6-dienyl)-2,4,6-trihydroxyphenyl)pentan-1-one (**3e**). Yellowish brown solid, yield 10.3%. ^{1}H-NMR (500 MHz, Methanol-d_4) d 5.90 (s, 1H), 5.13–5.19 (m, 1H), 5.04 (br. s., 1H), 3.17 (d, J = 6.85 Hz, 2H), 3.03 (t, J = 1.00 Hz, 2H), 2.03–2.04 (m, 2H), 1.91–1.94 (m, 2H), 1.73 (s, 3H), 1.61–1.67 (m, 2H), 1.60 (s, 3H), 1.54 (s, 3H), 1.38–1.41 (m, 2H), 0.94 (t, J = 7.34 Hz, 3H). ^{13}C-NMR (126 MHz, Methanol-d_4) 206.3, 163.5, 156.8, 133.3, 130.6, 124.1, 123.2, 43.2, 39.5, 27.2, 26.3, 24.4, 22.3, 20.7, 16.3, 14.8, 12.9. DIP: m/z 346.20.

Figure S27. ^{1}H-NMR spectrum of (*E*)-1-(3-(3,7-dimethylocta-2,6-dienyl)-2,4,6-trihydroxyphenyl)pentan-1-one.

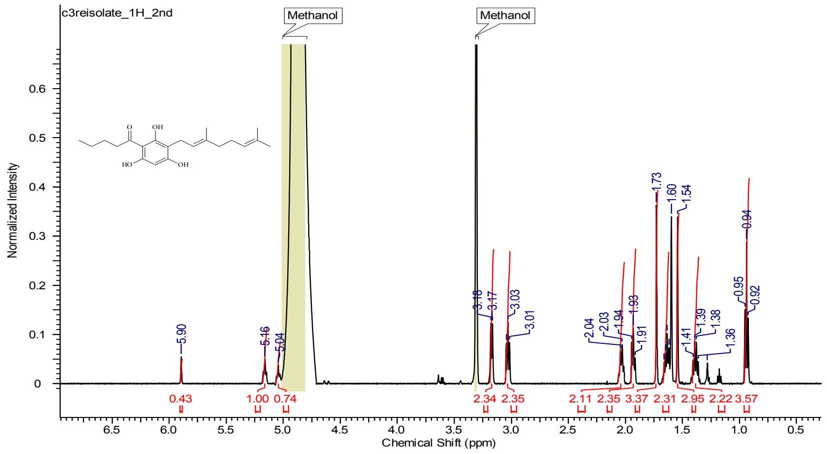

Figure S28. ^{13}C-NMR spectrum of (*E*)-1-(3-(3,7-dimethylocta-2,6-dienyl)-2,4,6-trihydroxyphenyl)pentan-1-one.

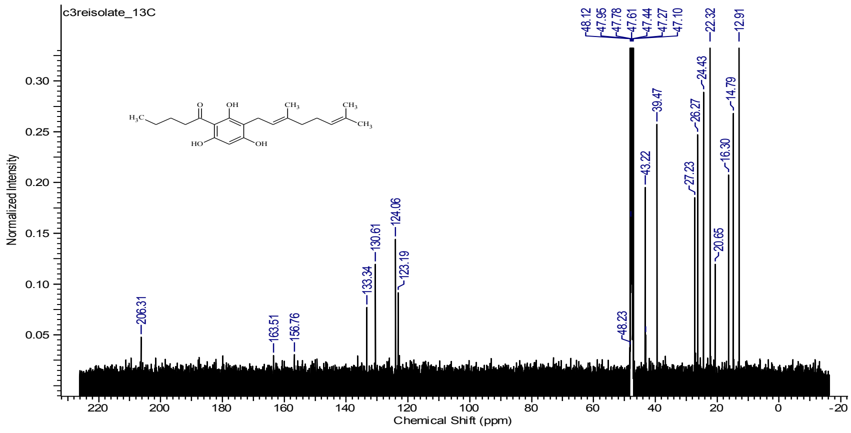

Figure S29. Mass spectrum of (*E*)-1-(3-(3,7-dimethylocta-2,6-dienyl)-2,4,6-trihydroxyphenyl)pentan-1-one.

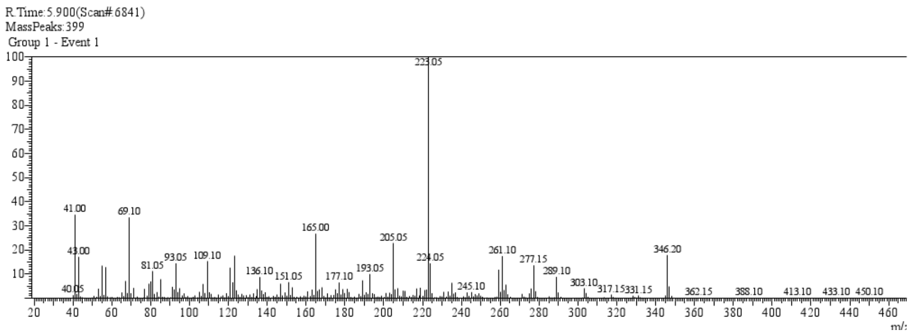

(E)-Cyclohexyl (3-(3,7-dimethylocta-2,6-dienyl)-2,4,6-trihydroxyphenyl)-methanone (**3f**). Brown solid, yield 5.7%. ^{1}H-NMR (500 MHz, Methanol-d_4) d 5.89 (s, 1H), 5.14–5.20 (m, 1H), 5.02–5.08 (m, 1H), 3.68–3.76 (m, 1H), 3.17 (d, *J* = 7.09 Hz, 2H), 2.04 (d, *J* = 7.34 Hz, 2H), 1.86–1.97 (m, 4H), 1.80 (d, *J* = 11.00 Hz, 2H), 1.73 (s, 3H), 1.60 (s, 3H), 1.55 (s, 3H), 1.31–1.44 (m, 4H), 1.23–1.30 (m, 2H). ^{13}C-NMR (126 MHz, Methanol-d_4) 209.4, 163.9, 161.9, 159.6, 133.2, 130.5, 124.1, 123.3, 106.8, 103.2, 93.6, 49.2, 39.5, 29.4, 26.3, 26.0, 25.9, 24.4, 20.7, 16.3, 14.8. DIP: *m/z* 372.05.

Figure S30. ^{1}H-NMR spectrum of (*E*)-cyclohexyl (3-(3,7-dimethylocta-2,6-dienyl)-2,4,6-trihydroxyphenyl)- methanone.

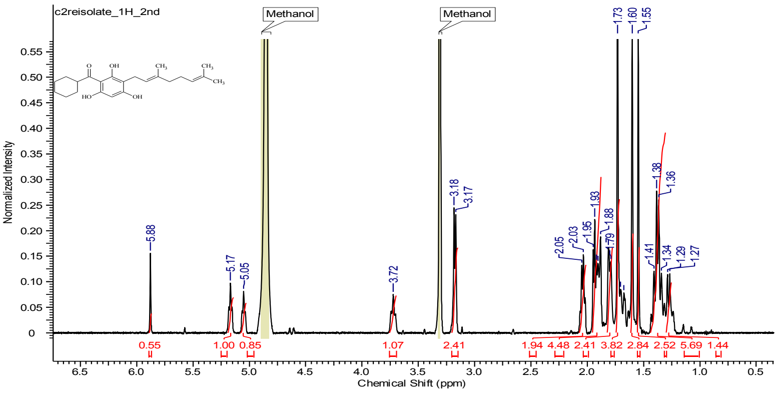

Figure S31. ^{13}C-NMR spectrum of (*E*)-cyclohexyl (3-(3,7-dimethylocta-2,6-dienyl)-2,4,6-trihydroxyphenyl)- methanone.

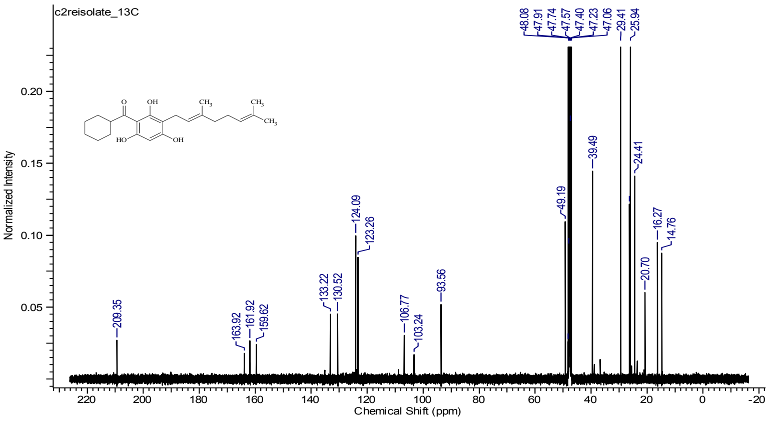

Figure S32. Mass spectrum of (*E*)-cyclohexyl (3-(3,7-dimethylocta-2,6-dienyl)-2,4,6-trihydroxyphenyl)- methanone.

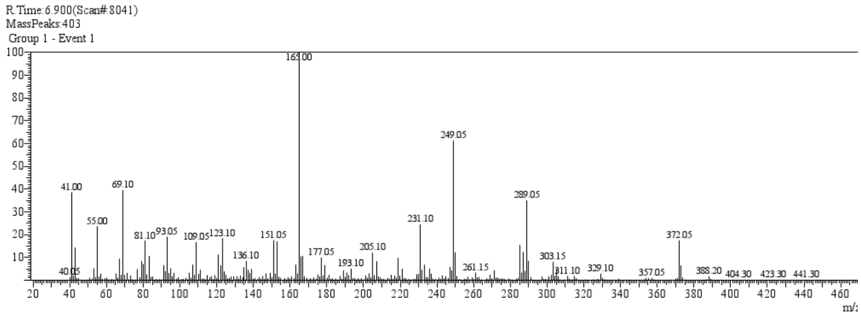

(E)-1-(3-((E)-3,7-Dimethylocta-2,6-dienyl)-2,4,6-trihydroxyphenyl)-3-phenylprop-2-en-1-one (**3g**). Light yellow solid, yield 11.7%. ^{1}H-NMR (500 MHz, Methanol-d$_4$) d 7.48-7.50 (m, 2H), 7.39-7.43 (m, 2H), 7.34–7.38 (m, 1H), 5.97 (s, 1H), 5.17–5.22 (m, 1H), 5.04–5.08 (m, 1H), 3.23 (d, J = 1.00 Hz, 2H), 3.03–3.11 (m, 1H), 2.73–2.79 (m, 1H), 2.02–2.08 (m, 2H), 1.92–1.98 (m, 2H), 1.75 (s, 3H), 1.62 (s, 3H), 1.56 (s, 3H). ^{13}C-NMR (126 MHz, Methanol-d$_4$) 195.8, 165.2, 161.1, 160.9, 139.2, 133.8, 130.6, 128.4, 128.2, 128.1, 125.9, 124.1, 122.6, 108.5, 101.7, 94.3, 78.9, 42.9, 39.5, 26.3, 24.4, 20.4, 16.3, 14.8. DIP: m/z 392.20.

Figure S33. ^{1}H-NMR spectrum of (*E*)-1-(3-((*E*)-3,7-dimethylocta-2,6-dienyl)-2,4,6-trihydroxyphenyl)-3-phenylprop2-en-1-one.

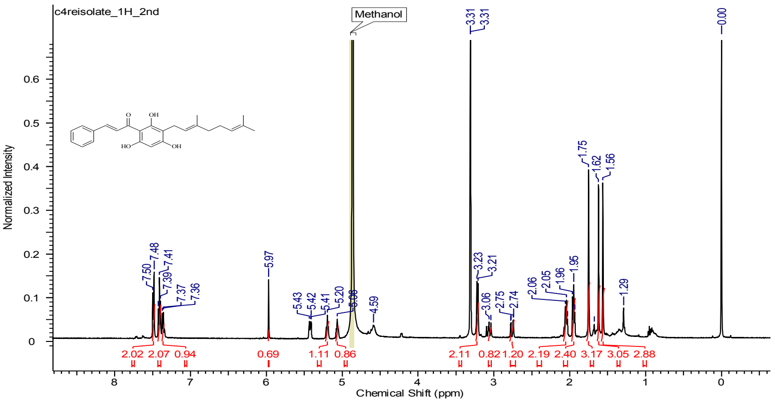

Figure S34. ^{13}C-NMR spectrum of (*E*)-1-(3-((*E*)-3,7-dimethylocta-2,6-dienyl)-2,4,6-trihydroxyphenyl)-3-phenylprop2-en-1-one.

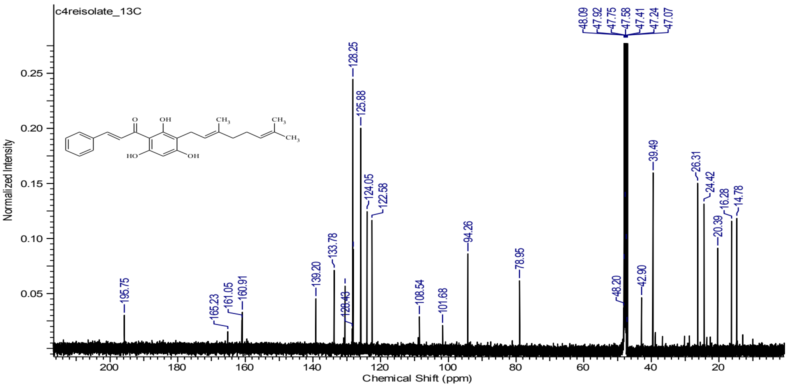

Figure S35. Mass spectrum of (*E*)-1-(3-((*E*)-3,7-dimethylocta-2,6-dienyl)-2,4,6-trihydroxyphenyl)-3-phenylprop2-en-1-one.

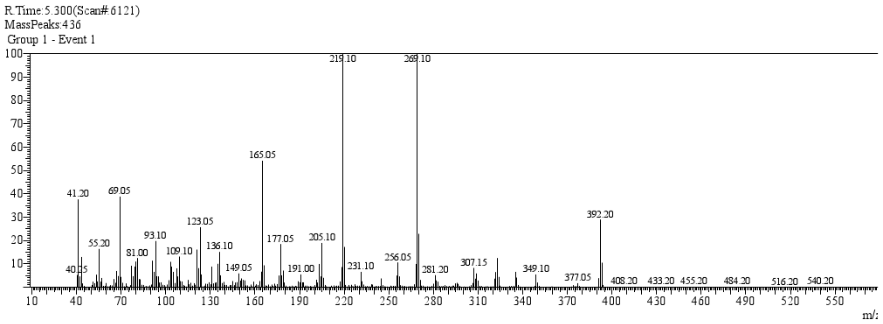

Figure S36. Three-dimensional (3-D) docking model of binding interaction of the compound **3g** with amino acid residues; the atom colouring for the compounds is the following: carbons in grey, oxygen in red, hydrogen in white and amino acid in green color. The green line indicates the hydrogen-bonding interactions.

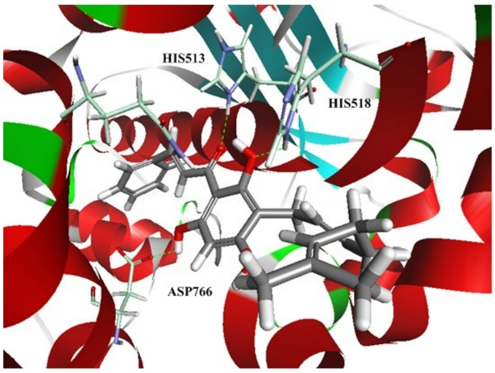

Figure S37. Three-dimensional (3-D) docking model of the superimposed structures of the active compound **3g** with the most active compound **3e** and also the (9*Z*,11*E*)-13(*R*)-hydroperoxy-9,11-octadecadienoic acid (13-HPOD) as LOX substrate. The blue color represents compound **3g**, red color represents compound **3e**, while yellow sticks represents 13-HPOD.

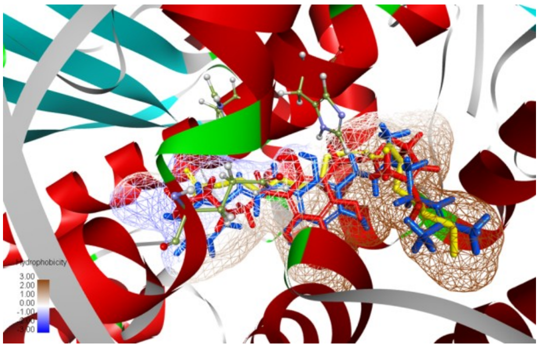

Figure S38. Three-dimensional (3-D) docking model of binding interaction of the NDGA with amino acid residues; the atom colouring for the compounds is the following: carbons in grey, oxygen in red, hydrogen in white and amino acid in green color. The orange line indicates the π-interaction.

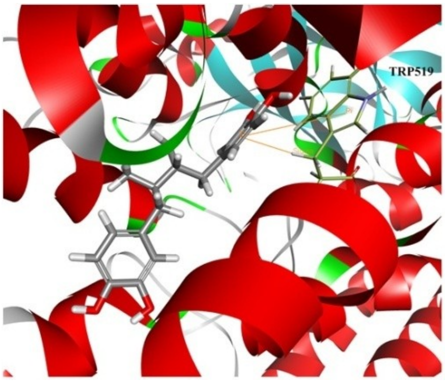

Supplement: Supplementary File 1 [file molecules-19-11645-s001.pdf]
